# Amygdala Volume Predicts Inter-Individual Differences in Fearful Face Recognition

**DOI:** 10.1371/journal.pone.0074096

**Published:** 2013-08-29

**Authors:** Ke Zhao, Wen-Jing Yan, Yu-Hsin Chen, Xi-Nian Zuo, Xiaolan Fu

**Affiliations:** 1 State Key Laboratory of Brain and Cognitive Science, Institute of Psychology, Chinese Academy of Sciences, Beijing, China; 2 University of Chinese Academy of Sciences, Beijing, China; 3 Key Laboratory of Behavior Science, Magnetic Resonance Imaging Research Center, Institute of Psychology, Chinese Academy of Sciences, Beijing, China; University Of Cambridge, United Kingdom

## Abstract

The present study investigates the relationship between inter-individual differences in fearful face recognition and amygdala volume. Thirty normal adults were recruited and each completed two identical facial expression recognition tests offline and two magnetic resonance imaging (MRI) scans. Linear regression indicated that the left amygdala volume negatively correlated with the accuracy of recognizing fearful facial expressions and positively correlated with the probability of misrecognizing fear as surprise. Further exploratory analyses revealed that this relationship did not exist for any other subcortical or cortical regions. Nor did such a relationship exist between the left amygdala volume and performance recognizing the other five facial expressions. These mind-brain associations highlight the importance of the amygdala in recognizing fearful faces and provide insights regarding inter-individual differences in sensitivity toward fear-relevant stimuli.

## Introduction

Fearful faces convey signals of potential threat, and recognizing such facial expressions with precision in conspecifics is evolutionarily advantageous [1], [2]. Within the human brain, the amygdala is presumed to play an essential role in processing such facial expressions [3]. Selective recognition deficits for fearful facial expressions were observed in humans with amygdala lesions [3]–[9]. A disrupted response to fearful faces in conspecifics was also shown in a recent study on amygdala-lesioned monkeys [10]. Converging evidence from functional neuroimaging studies indicated that viewing fearful faces led to increased activation in the amygdala for both normal humans [11]–[17] and monkeys [18].

Inter-individual differences in behavior can be predicted by differences in brain structures, providing insights into the neural substrates underlying the corresponding behaviors [19], [20]. Reduced or decreased amygdala volume has been observed in patients with spider phobia [21], posttraumatic stress disorder (PTSD) [22], [23], and pediatric anxiety (particularly social phobia) [24], compared to normal adults. These patients are thought to have an increased sensitivity to specific fear-related stimuli [21]–[25], such as patients with social phobia being more sensitive to critical facial expressions [26]. Research on normal subjects revealed that individuals with a smaller amygdala volume either had a smaller social network size [27] or were less extraverted [28]. Additionally, low extraversion/high introversion was related to increased levels of fear conditioning and fear sensitivity [29]. Hence, it is reasonable to hypothesize based on these studies of patients and normal adults that a smaller amygdala might be more sensitive to fear-relevant stimuli, such as fearful faces [30], and that performance when recognizing fearful faces could be predicted by the variation in volume between individuals.

To date, few studies have directly investigated the relationship between amygdala volume and inter-individual differences in fearful face recognition amongst a group of normal adults. In the present study, to evaluate an individual's performance in recognizing fearful facial expressions, a facial expression recognition test was conducted. To ensure the reliability of our behavioral data, we performed the same behavioral test offline twice with an interval of one month between tests. MRI scans were also obtained twice with a one-week interval to create a single, high signal-to-noise average volume [31]. We then calculated the correlation coefficients between amygdala volume and performance in fearful expression recognition. In addition, factors such as the intensity of fear in the presented images and the participants' trait anxiety levels were also considered [27], [32]–[35] to obtain a more comprehensive understanding of the relationship between amygdala volume and performance in fearful face recognition.

## Methods

### Ethics statement

The experimental procedure was approved by the IRB of the Institute of Psychology at the Chinese Academy of Sciences. All participants provided informed written consent before participating in our experiments.

### Participants

A total of 30 right-handed normal undergraduates (age: 20.93±1.72 yrs; 21 females) from Southwest University in China were recruited. Participants were asked about any severe physical or mental injuries they had in the past, prior to recruitment, and only those who reported no severe physical or mental injuries were recruited. In addition, before entering the MRI scanner, they completed a questionnaire provided by the Southwest University MRI Center that required all individuals to honestly report their current health status and medical records, including physical injuries and mental disorders. Of the 30 participants, two failed to submit their trait anxiety surveys through email, leaving 28 participants who completed all tests and surveys. One participant whose accuracy scores for recognizing fearful faces in both facial expression recognition tests were greater than 2.0 standard deviations from the overall mean score was excluded from all subsequent analyses (Test 1: 0.17<0.63 – 0.16*2 = 0.31; Test 2: 0.20<0.63 – 0.16*2 = 0.31). Another participant whose accuracy score for recognizing fearful faces in a single facial expression recognition test was more than 2.0 standard deviations from the overall mean score was also excluded from all subsequent analyses (Test 2: 0.97>0.63+0.16*2 = 0.95).

In addition, 34 adults (age: 22.71±1.38 yrs; 20 females) were recruited to evaluate the intensity of fear presented by the human model in each image. None of the 34 randomly recruited participants performed any other experiment in this study except for the 7-point scale survey to evaluate the intensity of fear in each image.

### Behavioral tests and measures

**Figure 1:**
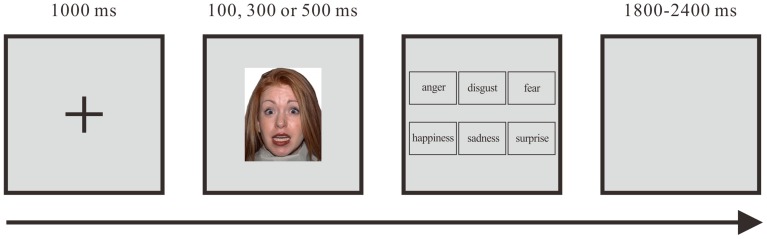
The procedure for a single trial of the facial expression test.

A 17-inch cathode-ray tube (CRT) monitor running at a refresh rate of 60 Hz and the software package E-prime 2.0 were used for stimuli presentation and data collection. The target stimuli were images of six types of facial expressions (anger, disgust, fear, happiness, sadness, and surprise) posed by human models (5 males and 5 females from different ethnic backgrounds, to account for the effect between perceived race and the race of the subject [36]) from the NimStim database [37]. A total of 60 images (one image for each of the 6 basic expressions ×10 models) were selected from the database and trimmed to 192×220 pixels. All stimuli were presented on a uniform silver gray background, which remained silver gray throughout the experiment. The protocol was based on Ekman and Friesen's Brief Affect Recognition Test (BART) [38], with a few minor modifications. In a single trial, a black fixation cross was first presented in the center of the silver gray background for 1000 ms, followed by a facial expression image presented in the center of the screen for either 100, 300 or 500 ms. Six emotion options (anger, disgust, fear, happiness, sadness and surprise) were then presented on the screen. Subjects were asked to choose the emotion option that best describes the facial expression. After participants chose an answer, an inter-trial interval ranging from 1800 ms to 2400 ms was randomly chosen and displayed in between each trial (Figure 1). Participants performed 60 trials per display duration (100, 300 or 500 ms) for a total of 180 trials (60 trials ×3 display durations) in a single test. Each participant completed 2 tests (360 trials) with an interval of one month between the two tests for reliability. Prior to the formal experiment, subjects were required to perform 10 trials to become familiar with the procedures and tasks.

To measure trait anxiety, we contacted the participants through email and asked them to complete the State-Trait Anxiety Inventory (STAI). The STAI is commonly used to measure state-trait anxiety [39]. The trait anxiety subscale contains 20 items rated on a 4-point scale (e.g., from “Almost Never” to “Almost Always”). Higher scores indicate greater anxiety.

To account for the intensity of fear in each image, we conducted a separate survey. The fearful facial expression images of 10 different models selected from the NimStim database (NimStim database individual IDs: 01, 07, 11, 17, 19, 27, 34, 38, 42, and 43) used in our facial expression recognition test were listed in a questionnaire. The subjects were 34 randomly recruited Chinese adults whose task was to evaluate the intensity of fear presented by the human model in each image on a 7-point scale (“1” represents the lowest intensity of fear and “7” represents the highest intensity of fear). After acquiring a mean intensity score for each fearful expression image, we sorted the 10 images according to their scores from the lowest to the highest intensity. The three lowest-intensity images were then categorized as the low-intensity group, and the three highest-intensity images were categorized as the high-intensity group (see Table S1 in File S1).

### Structural MRI data acquisition and morphometric analysis

During the MRI scans, two high-resolution structural images of the whole brain were acquired for each participant on a Siemens 3T scanner (Siemens, Erlangen, Germany) with a 12-channel head matrix coil. Structural MRI data for the sample were acquired using sagittal T1-weighted magnetization prepared rapid gradient echo (MPRAGE) sequences (TI = 1100 ms, TR/TE = 2530/2.5 ms, FA = 7°, FOV  = 256×256 mm^2^, voxel-size  = 1.0×1.0×1.3 mm^3^, 128 slices).

All structural image analysis was conducted using the Connectome Computation System (CCS: http://lfcd.psych.ac.cn/ccs.html) pipeline [40]. Specifically, each participant's MR images were first denoised through a spatially adaptive non-local means filter [41], [42]. The two denoised MRI scans for each participant were averaged to create a single high signal-to-noise average volume [31]. To determine the amygdala volume, we performed quantitative morphometry analysis on the averaged T1-weighted MRI data using an automated segmentation and probabilistic region of interest (ROI) labeling technique [43]. These images were first corrected for intensity variations due to MR inhomogeneities [44]. As described in the Freesurfer Wiki document (http://surfer.nmr.mgh.harvard.edu/fswiki), a hybrid watershed/surface deformation procedure [45] was first employed to extract brain tissues that were then automatically segmented into the cerebrospinal fluid (CSF), white matter (WM) and deep gray matter (GM) volumetric structures [44]. To explore the relationship between fearful face recognition performance and other brain areas, we further conducted individual cortical surface reconstructions to measure the volumes of these regions. Two researchers (Y.M and L.Y.), blind to the hypotheses, manually inspected the results of the automated brain tissue segmentation. The criteria used for quality assurance on brain extraction, surface reconstruction and anatomical image registration are as follows: 1) the quality of the brain extraction and intensity bias correction must be visually assessed and manually corrected if the procedure failed, and 2) the brain tissue segmentation was also visually checked to ensure good quality. A detailed description of the criteria can be found on the following website: http://lfcd.psych.ac.cn/ccs/QC.html, and in our previous work [40]. After the visual inspection, there were 6 participants whose brainmask datasets were manually edited to achieve better estimates of pial surfaces. The results of the automated segmentation were verified as accurate without the need for any correction.

The cortical surface was parcellated into 34 parcellation elements (parcels) for each hemisphere, defined by the Desikan-Killiany atlas in FreeSurfer [46], [47]. The subcortical structure was segmented into a total of 17 regions, consisting of the Brainstem and 8 regions in each hemisphere: amygdala, caudate, hippocampus, accumbens-area, pallidum, putamen, thalamus-proper and cerebellum-cortex. The volume of each of these 41 brain regions was then calculated for subsequent exploratory analyses.

### Mind-Brain Association Analyses

We employed linear regression models to examine the relationships between both left and right amygdala volume (independent variables) and the accuracy (number of correct judgments/total number of judgments) for recognizing fearful faces (dependent variable). To control for the total intracranial volume (ICV), we included the ICV as a covariate in the regression model. Instead of dividing the amygdala volume by the total intracranial volume (ICV), we calculated the correlation coefficients between the behavioral data and amygdala volume using a linear regression model with the ICV as a covariate to control for the inter-individual variability in brain size. This approach was used because the reliability theory of measurements states that the ratio measurement has reduced test-retest reliability [48], and the approach was inspired by our recent demonstration of the standardization of functional connectome metrics [49]. We adopted this approach in the following analysis and calculated the power for each correlation coefficient. To demonstrate the differences in detecting mind-brain associations between the two approaches, we also included results with the amygdala volume divided by the ICV (Table S2 in File S1). To further examine a key concept for mind-brain association studies – test-retest reliability – we computed the intraclass correlation coefficients (ICCs) for both the amygdala volume and the facial expression test. Further exploratory analyses examined the relationships between fearful face recognition performance and the volumes of the 41 (7 subcortical and 34 cortical) other brain regions for each hemisphere.

## Results

**Figure 2:**
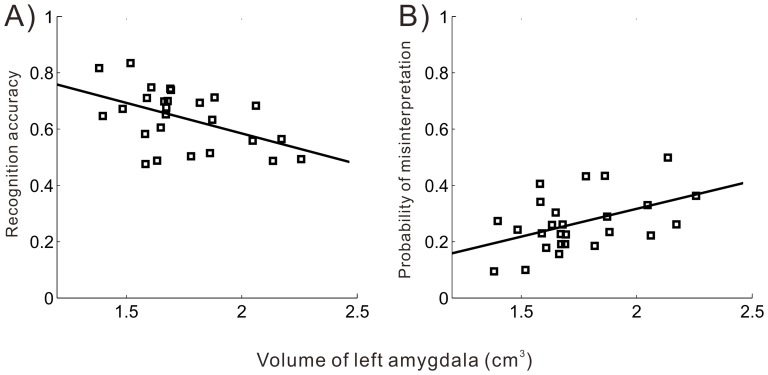
The left amygdala volume was correlated with the mean accuracy for recognizing fearful faces and the mean error rates for judging fear as surprise. A) Scatter plot of recognition accuracy for fearful facial expressions (y-axis) versus the total adjusted left amygdala volume (x-axis, cm3). B) Scatter plot of the probability of misinterpreting fear as surprise (y-axis) versus the total adjusted left amygdala volume (x-axis, cm3). The best-fit lines are plotted based on the average results of the two experiments. The correlation coefficients between amygdala volume and performance in fearful face recognition were obtained while controlling for total intracranial volume.

Our results show that the volume of the left amygdala negatively correlated with the subjects' accuracy on fearful face recognition in Test 1 (*r* = −0.61, *p* = 0.001, power  = 0.94), Test 2 (*r* = −0.43, *p* = 0.03, power  = 0.66), and the average of the two tests (*r* = −0.66, *p*<0.001, power  = 0.98) (Figure 2 and Table 1). The correlation coefficients for the recognition accuracy score and left amygdala volume for Test 1 and Test 2 (through Fisher's z-Test) did not differ (*z* = 1.25, *p* = 0.10). Therefore, data from Tests 1 and 2 were merged and mean scores were used to conduct all subsequent analyses in the present study. To further investigate the specificity of the relationship, a total of 36 outcomes (6 presented facial expressions and 6 emotion options) were generated in every subject's confusion matrix [50], indicating how subjects judged the presented facial expressions. Relationships between the presented facial expressions (ground truth) and the participants' judgments (classification results) are shown in detail in Table 2. We did not find any other consistent correlations (in Test 1 or Test 2) between amygdala volume and recognition performance for the other facial expressions (Table S3 in File S1). Interestingly, we discovered that the left amygdala volume is positively correlated with the probability of misinterpreting fearful faces as surprised faces in Test 1 (*r* = 0.57, *p* = 0.003, power  = 0.87), Test 2 (*r* = 0.45, *p* = 0.026, power  = 0.66), and the average of the two tests (*r* = 0.63, *p* = 0.001, power  = 0.90).

**Table 1:** Correlation based on linear regression using amygdala and hippocampal volumes as the independent variables and performance in recognizing fearful faces as the dependent variable.

	Amygdala		Hippocampus	
	Left	Right	Left	Right
**Test 1**				
fear-fear	**−0.606 (0.001)**	−0.270 (0.192)	−0.360 (0.077)	−0.278 (0.178)
fear-surprise	**0.571 (0.003)**	−0.051 (0.808)	0.299 (0.147)	0.231 (0.267)
**Test 2**				
fear-fear	−**0.432 (0.031)**	−0.302 (0.142)	−0.194 (0.352)	−0.116 (0.581)
fear-surprise	**0.445 (0.026)**	0.271 (0.191)	0.217 (0.297)	0.079 (0.709)
**Mean**				
fear-fear	−**0.663 (0.000)**	−0.372 (0.067)	−0.350 (0.086)	−0.246 (0.235)
fear-surprise	**0.625 (0.001)**	0.212 (0.310)	0.316 (0.124)	0.184 (0.378)

The table shows correlation coefficients (p-values). Results with p-values <0.05 are indicated in bold. The correlation coefficients between amygdala volume and performance in fearful face recognition were obtained while controlling for total intracranial volume.

**Table 2:** The confusion matrix of relationships between the presented facial expressions and the participants' judgments.

Presented facial expression	Identified facial expression					
	Anger	Disgust	Fear	Happiness	Sadness	Surprise
Anger	**0.69**	0.25	0.01	0.00	0.04	0.01
Disgust	0.25	**0.65**	0.04	0.00	0.04	0.02
Fear	0.01	0.03	**0.64**	0.00	0.05	0.27
Happiness	0.00	0.00	0.00	**0.99**	0.00	0.00
Sadness	0.02	0.14	0.01	0.00	**0.77**	0.06
Surprise	0.00	0.00	0.20	0.00	0.00	**0.80**

The data presented in each column is the probability of the participants' judgments. The recognition accuracy for each facial expression is marked in bold. All trials from Tests 1 and 2 are included.

Our exploratory analyses showed that no correlation was significant in both tests for any other brain regions, even with a lenient threshold (uncorrected *p*<0.05). No cortical or subcortical region showed consistent significant correlations with fearful face recognition accuracy except for the left amygdala (for more details see Tables S4-6 in File S1). We also showed the distribution of amygdala volume for subcortical regions across two tests (Tables S7-10 in File S1).

In addition, to determine whether differences existed in the correlation coefficients for amygdala volume and fearful face recognition across the 3 image display durations (100 ms: *r* = −0.45, *p* = 0.026, power  = 0.66; 300 ms: *r* = −0.51, *p* = 0.009, power  = 0.79, and 500 ms: *r* = −0.49, *p* = 0.014, power  = 0.76), we used Fisher's z-Test. The results showed that the correlation coefficients between recognition accuracy score and left amygdala volume for the display durations of 100 ms compared with 300 ms (*z* = 0.390, *p* = 0.35) and 500 ms (*z* = 0.256, *p* = 0.40) were not significant; 300 ms and 500 ms (*z* = −0.133, *p* = 0.55) showed no difference as well. To examine whether the intensity of fear in the images we selected had an effect on the relationship between the amygdala volume and fearful face recognition, a Fisher's z-Test was conducted on the accuracy-volume correlation coefficients derived from the low-intensity image group and the high-intensity image group. Analysis of the intensity of fear in the images revealed that the accuracy-volume correlation coefficients of the high-intensity group (*r* = −0.63, *p* = 0.001, power  = 0.90) were marginally higher than those in the low-intensity group (*r* = −0.41, *p* = 0.04, power  = 0.55), (*z* = 1.53, *p* = 0.06). Our results revealed no correlation between amygdala volume and trait anxiety (left: *r* = −0.13, *p* = 0.54, power  = 0.09; right *r* = −0.007, *p* = 0.974, power <0.05). The test-retest reliability for the left amygdala volume, measured by the intraclass correlation coefficient (ICC), was 0.810, and the test-retest reliability for the right amygdala volume was 0.734. The test-retest reliability for the facial expression test was 0.757.

## Discussion

Numerous studies have observed that the amygdala is particularly responsive to fearful facial expressions [8]–[9], [11]–[18], [51]–[54], yet few studies have investigated the relationship between amygdala volume and inter-individual differences in performance on tests of fearful face recognition. To our knowledge, this may be the first study that reveals an association between amygdala volume and fearful face recognition amongst normal adult subjects. Our results revealed that the left amygdala volume negatively correlates with recognition accuracy for fearful faces. Additionally, the left amygdala volume positively correlated with the probability of misrecognizing expressions of fear as surprise. These findings were based on data obtained from two behavioral experiments and two MRI scans conducted on each individual. Test-retest reliability was almost perfect (ICC = 0.810) for the left amygdala volume and was substantial for the right amygdala volume (ICC = 0.734) [55]. Exploratory analyses revealed that only performance for recognizing fearful facial expressions correlated with left amygdala volume across both tests. Such a relationship did not exist between the left amygdala volume and the performance for recognizing the other five facial expressions. Further analysis revealed that the correlation with performance for recognizing fearful facial expressions did not exist for any subcortical or cortical regions except the left amygdala volume across both tests.

This specific relationship between amygdala volume and fearful face recognition suggests a crucial role for the amygdala in the processing of fearful faces. In previous studies, researchers found that patients with amygdala lesions had impaired recognition performance [8]. Increased amygdala activation was also observed in both humans [11]–[17] and monkeys [18] when processing fearful facial expressions compared with other facial expressions. The present study provides further insights regarding the amygdala and its relationship to fearful face recognition, demonstrating that inter-individual differences in amygdala volume can predict performance on tests of fearful face recognition.

Amongst facial expressions, fearful faces might not only be hard to recognize [5]–[6], [56]–[57] but also have the highest probability of being misinterpreted as surprised faces [5], [58]–[61]. Consistent with these studies, our behavioral results indicated that the accuracy scores for recognizing fearful faces were the lowest. Participants were more inclined to misinterpret fear as surprise. Confusion of facial expressions between fear and surprise is universal across cultures [62]. This might be because both surprised faces and fearful faces are “wide-eyed, information gathering” facial expressions. In addition, this confusion is one-sided and recognition accuracy for surprised faces is generally slightly higher than fearful faces, as shown in previous studies [5], [61], [63]. Surprised faces are rarely misinterpreted as any other emotions except fear, whilst fearful faces are generally misinterpreted as surprise but also misinterpreted as anger, disgust, and sadness. Furthermore, through Mind-Brain association analysis, our results indicated that smaller amygdala volumes are associated with better performance for recognizing fearful faces (overall made less mistakes), whilst individuals with larger amygdala volumes were more inclined to misinterpret fearful faces as surprise. However, no relationship was found between amygdala volume and recognition accuracy for surprised faces or the probability of misrecognizing surprised faces as fear.

Therefore, an intriguing question begs an answer; “why does a smaller amygdala predict better performance in fearful face recognition?” Our findings, from the relationship between amygdala volume and fearful face recognition to the one-sided confusion of misinterpreting fearful faces as surprise, may speak to this. Our results support the speculation that the amygdala responds most specifically to fear when subjects attend to the stimuli [64], [65] and are highly sensitive to fearful faces, as demonstrated in studies showing greater amygdala activation for fearful faces in comparison to angry faces [66], happy faces [11], and neutral faces [12], [13]. Therefore, it is possible that subjects with smaller amygdala volumes are more sensitive to fear-relevant stimuli, and these subjects had higher accuracy scores in fearful face recognition. However, subjects with larger amygdala volumes were less sensitive to fear-relevant stimuli and had a higher probability of misrecognizing fear as surprise. This speculation is also partly supported by findings from previous studies, which observed reduced or decreased amygdala volume in patients with spider phobia [21], posttraumatic stress disorder (PTSD) [22], [23], or pediatric anxiety (particularly social phobia) [24]. These patients were thought to have an increased sensitivity to specific fear-relevant stimuli [21]–[25]. Together with observations from lesion studies of patients with amygdala damage (especially SM), these studies highlight the indispensable role that the amygdala plays in promoting survival by compelling the organism away from danger [67], [68], and it appears that without the amygdala, the evolutionary value of fear is lost [69].

Our results reveal that the intensity of the fearful facial expressions and trait anxiety did not influence the relationship between amygdala volume and fearful face recognition. This is consistent with several previous findings that found brain regions other than the amygdala, such as the left anterior insula, left pulvinar and right anterior cingulate, to be responsive to increasing intensity of fear [14], [70].

Several limitations should be noted when interpreting our findings. Our study has shown an association between amygdala volume and fearful face recognition performance using a small sample. While we utilized the test-retest measure to examine the reproducibility of our findings in this small sample, future work based on a large sample would further verify these findings and could examine gender effects on this mind-brain association. In addition, it is relatively difficult to achieve an accurate segmentation of the amygdala, compared to other brain structures, because of its small size. However, the test-retest reliability for the left amygdala volume was almost perfect (ICC = 0.810), indicating robust and reliable amygdala segmentation in our study.

## Supporting Information

File S1 Tables S1–S10.
(DOCX)
