# Supplementary material for: Amygdala Volume Predicts Inter-Individual Differences in Fearful Face Recognition
Source: PLoS One. 2013 Aug 29;8(8):e74096. doi: 10.1371/journal.pone.0074096 (PMC3756978; doi:10.1371/journal.pone.0074096)
Supplement: File S1 — Tables S1–S10. (DOCX) [file pone.0074096.s001.docx]

**Supplementary Materials**

**Table S1.** The mean intensity score of the 10 fearful facial expression images selected from the NimStim Database.

| Image ID | Fear face | Degree of intensity | Image ID | Fear face | Degree of intensity |
| --- | --- | --- | --- | --- | --- |
| 07 |  | 3.66 + 1.47 | 27 |  | 4.89 + 1.60 |
| 01 | 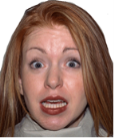 | 3.80 + 1.47 | 43 |  | 5.06 + 1.91 |
| 17 |  | 4.17 + 1.58 | 11 |  | 5.11 + 1.51 |
| 42 |  | 4.74 + 1.48 | 34 |  | 5.74 + 1.57 |
| 38 |  | 4.88 + 1.74 | 19 |  | 6.48 + 0.92 |

Images of model ID #1 (who has provided consent) are shown in the table as an example. Images of other models (ID: 07, 11, 17, 19, 27, 34, 38, 42, and 43) can be found in the NimStim Database.

**Table S2.** Correlation based on linear regression using amygdala and hippocampal volumes as the independent variables and performance in recognizing fear faces as the dependent variable.

|  | Amygdala | | Hippocampus | |
| --- | --- | --- | --- | --- |
|  | Left | Right | Left | Right |
| **Test 1** |  |  |  |  |
| fear-fear | **-0.49 (0.013)** | -0.30 (0.144) | -0.39 (0.053) | -0.21 (0.315) |
| fear-surprise | **0.49 (0.013)** | -0.10 (0.637) | 0.36 (0.082) | 0.21 (0.322) |
| **Test 2** |  |  |  |  |
| fear-fear | -0.34 (0.097) | -0.28 (0.171) | -0.16 (0.442) | -0.05 (0.803) |
| fear-surprise | **0.42 (0.036)** | 0.27 (0.189) | 0.23 (0.266) | 0.10 (0.621) |
| **Mean** |  |  |  |  |
| fear-fear | **-0.53 (0.006)** | -0.38 (0.060) | -0.35 (0.089) | -0.16 (0.441) |
| fear-surprise | **0.57 (0.003)** | 0.25 (0.239) | 0.36 (0.079) | 0.19 (0.371) |

The table shows correlation coefficients (p-values). Results with p-values < 0.05 are indicated in bold. Volumes were divided by the total intracranial volume.

|  | Test 1 | | Test 2 | | Mean | |
| --- | --- | --- | --- | --- | --- | --- |
|  | L-amyg | R-amyg | L-amyg | R-amyg | L-amyg | R-amyg |
| an-an | -0.022 (0.918) | -0.146 (0.486) | -0.108 (0.607) | -0.186 (0.373) | -0.074 (0.726) | -0.183 (0.380) |
| an-di | 0.056 (0.790) | 0.194 (0.352) | 0.045 (0.830) | 0.191 (0.360) | 0.056 (0.790) | 0.215 (0.302) |
| an-fe | -0.086 (0.684) | -0.179 (0.391) | 0.189 (0.365) | 0.189 (0.365) | 0.035 (0.870) | -0.029 (0.890) |
| an-ha | -0.241 (0.246) | 0.009 (0.965) | -0.042 (0.842) | -0.165 (0.431) | -0.18 (0.388) | -0.132 (0.529) |
| an-sa | -0.225 (0.279) | -0.093 (0.658) | 0.082 (0.696) | -0.033 (0.877) | -0.065 (0.757) | -0.073 (0.730) |
| an-su | 0.306 (0.136) | 0.103 (0.624) | 0.552 (0.004)****** | 0.161 (0.443) | 0.421 (0.036)* | 0.133 (0.527) |
| di-an | 0.239 (0.249) | 0.219 (0.292) | 0.331 (0.106) | 0.014 (0.946) | 0.356 (0.081) | 0.135 (0.520) |
| di-di | -0.373 (0.066) | -0.399 (0.048)* | -0.398 (0.049)***** | -0.144 (0.492) | -0.498 (0.011)* | -0.343 (0.093) |
| di-fe | 0.119 (0.572) | 0.291 (0.157) | -0.271 (0.189) | -0.077 (0.714) | -0.059 (0.780) | 0.162 (0.441) |
| di-ha | 0.028 (0.893) | 0.181 (0.387) | 0.064 (0.762) | 0.120 (0.567) | 0.060 (0.775) | 0.219 (0.293) |
| di-sa | 0.195 (0.350) | 0.154 (0.461) | 0.390 (0.054) | 0.337 (0.099) | 0.369 (0.069) | 0.309 (0.133) |
| di-su | 0.209 (0.316) | 0.196 (0.349) | 0.182 (0.385) | 0.374 (0.065) | 0.257 (0.215) | 0.354 (0.082) |
| fe-an | 0.076 (0.719) | -0.072 (0.731) | -0.199 (0.340) | -0.182 (0.384) | 0.001 (0.995) | -0.110 (0.601) |
| fe-di | 0.065 (0.757) | 0.331 (0.107) | 0.045 (0.830) | 0.043 (0.837) | 0.076 (0.720) | 0.270 (0.1920) |
| fe-fe | -0.606 (0.001)** | -0.27 (0.192) | -0.432 (0.031)* | -0.302 (0.142) | -0.663 (0.000)** | -0.372 (0.067) |
| fe-ha | N/A | N/A | N/A | N/A | N/A | N/A |
| fe-sa | 0.077 (0.713) | 0.404 (0.045)* | 0.079 (0.709) | 0.148 (0.480) | 0.090 (0.670) | 0.293 (0.155) |
| fe-su | 0.571 (0.003)** | 0.051 (0.808) | 0.445 (0.026)* | 0.271 (0.191) | 0.625 (0.001)** | 0.212 (0.310) |
| ha-an | 0.149 (0.477) | 0.188 (0.369) | 0.053 (0.802) | -0.073 (0.730) | 0.160 (0.446) | 0.138 (0.510) |
| ha-di | 0.058 (0.785) | 0.024 (0.910) | -0.240 (0.249) | -0.054 (0.796) | -0.098 (0.640) | -0.013 (0.949) |
| ha-fe | 0.149 (0.477) | 0.188 (0.369) | N/A | N/A | 0.149 (0.477) | 0.188 (0.369) |
| ha-ha | -0.139 (0.509) | -0.206 (0.324) | 0.135 (0.521) | 0.091 (0.665) | -0.101 (0.632) | -0.178 (0.394) |
| ha-sa | 0.133 (0.527) | 0.331 (0.106) | N/A | N/A | 0.133 (0.527) | 0.331 (0.106) |
| ha-su | N/A | N/A | N/A | N/A | N/A | N/A |
| sa-an | 0.460 (0.021)***** | 0.487 (0.014)***** | 0.250 (0.228) | 0.198 (0.343) | 0.378 (0.062) | 0.358 (0.079) |
| sa-di | 0.204 (0.328) | 0.160 (0.445) | -0.115 (0.585) | 0.023 (0.912) | 0.087 (0.679) | 0.131 (0.533) |
| sa-fe | 0.281 (0.173) | 0.064 (0.763) | -0.224 (0.283) | -0.207 (0.321) | 0.03 (0.888) | -0.090 (0.668) |
| sa-ha | -0.073 (0.729) | -0.160 (0.444) | N/A | N/A | -0.073 (0.729) | -0.160 (0.444) |
| sa-sa | -0.380 (0.061) | -0.241 (0.245) | -0.066 (0.754) | -0.112 (0.593) | -0.294 (0.154) | -0.228 (0.274) |
| sa-su | 0.156 (0.458) | 0.001 (0.996) | 0.241 (0.246) | 0.147 (0.483) | 0.232 (0.264) | 0.089 (0.673) |
| su-an | 0.013 (0.953) | -0.074 (0.726) | -0.073 (0.729) | -0.16 (0.444) | -0.042 (0.842) | -0.165 (0.431) |
| su-di | 0.219 (0.292) | 0.174 (0.406) | 0.244 (0.239) | 0.463 (0.020)***** | 0.330 (0.108) | 0.454 (0.023)***** |
| su-fe | -0.147 (0.482) | 0.012 (0.956) | -0.271 (0.190) | -0.298 (0.148) | -0.257 (0.216) | -0.161 (0.442) |
| su-ha | 0.023 (0.913) | 0.012 (0.955) | 0.139 (0.506) | 0.263 (0.204) | 0.123 (0.558) | 0.208 (0.317) |
| su-sa | N/A | N/A | 0.089 (0.673) | 0.239 (0.250) | 0.089 (0.673) | 0.239 (0.250) |
| su-su | 0.127 (0.544) | -0.019 (0.928) | 0.244 (0.241) | 0.237 (0.255) | 0.224 (0.283) | 0.115 (0.583) |

**Table S3.** Correlation coefficients between the amygdala volume (left and right) and each choice in the confusion matrix for Test 1, Test 2 and the average of two tests (Mean).

The values in the table indicate correlation coefficients (and p-value in brackets) between the amygdala volume and each emotion choice while controlling for total intracranial volume (ICV). The “fe-fe” means that participants identify fear faces as fear faces. “L-amyg” means the volume of left amygdala, while “R-amyg” means the volume of right amygdala. Abbreviations are defined as follow: an = anger, di = disgust, fe = fear, ha = happiness, sa = sad, su = surprise. * indicates p < 0.05, and ** indicates p < 0.01. N/A indicated the correlation coefficient is considered as irrelevant because ratio of misrecognition is less than 0.03.

**Table S4.** The correlation coefficients for the relationship between the volume of the left hemispheric regions and fear-fear (fear-surprise) choice in Test 1, Test 2 and the average of two tests.

| Left Hemisphere | fe-fe1 | fe-su1 | fe-fe2 | fe-su2 | fear_mean | fe-su_mean |
| --- | --- | --- | --- | --- | --- | --- |
| lh_bankssts | -0.306 (0.136) | 0.253 (0.222) | -0.113 (0.591) | 0.105 (0.618) | -0.261 (0.208) | 0.214 (0.304) |
| lh_caudalanteriorcingulate | -0.264 (0.202) | 0.318 (0.122) | 0.200 (0.338) | -0.164 (0.433) | -0.016 (0.939) | 0.070 (0.741) |
| lh_caudalmiddlefrontal | -0.265 (0.200) | 0.117 (0.576) | 0.067 (0.749) | -0.167 (0.426) | -0.110 (0.601) | -0.046 (0.828) |
| lh_cuneus | -0.512 (0.009)** | 0.510 (0.009)****** | 0.147 (0.484) | -0.130 (0.537) | -0.200 (0.338) | 0.202 (0.333) |
| lh_entorhinal | -0.398 (0.049)* | 0.198 (0.343) | -0.161 (0.443) | 0.021 (0.922) | -0.349 (0.087) | 0.126 (0.547) |
| lh_fusiform | -0.233 (0.263) | 0.279 (0.177) | -0.033 (0.875) | 0.091 (0.666) | -0.161 (0.442) | 0.220 (0.292) |
| lh_inferiorparietal | -0.253 (0.223) | 0.233 (0.263) | 0.089 (0.671) | -0.07 (0.738) | -0.087 (0.68) | 0.085 (0.687) |
| lh_inferiortemporal | -0.370 (0.069) | 0.300 (0.146) | 0.266 (0.198) | -0.269 (0.193) | -0.032 (0.88) | -0.012 (0.956) |
| lh_isthmuscingulate | -0.219 (0.293) | 0.391 (0.053) | 0.096 (0.648) | -0.082 (0.696) | -0.062 (0.768) | 0.167 (0.426) |
| lh_lateraloccipital | -0.246 (0.236) | 0.289 (0.161) | 0.213 (0.306) | -0.180 (0.390) | 0.004 (0.985) | 0.043 (0.839) |
| lh_lateralorbitofrontal | -0.170 (0.416) | 0.063 (0.765) | -0.090 (0.670) | -0.076 (0.72) | -0.164 (0.434) | -0.015 (0.942) |
| lh_lingual | 0.061 (0.771) | -0.149 (0.478) | 0.102 (0.626) | -0.268 (0.194) | 0.108 (0.606) | -0.266 (0.199) |
| lh_medialorbitofrontal | -0.204 (0.329) | 0.279 (0.178) | -0.293 (0.155) | 0.299 (0.147) | -0.327 (0.111) | 0.360 (0.077) |
| lh_middletemporal | -0.132 (0.528) | 0.110 (0.601) | 0.190 (0.364) | -0.195 (0.349) | 0.055 (0.795) | -0.07 (0.741) |
| lh_parahippocampal | -0.419 (0.037)***** | 0.265 (0.201) | -0.362 (0.075) | 0.246 (0.236) | -0.503 (0.01)***** | 0.316 (0.124) |
| lh_paracentral | 0.042 (0.841) | 0.033 (0.876) | -0.044 (0.836) | 0.130 (0.535) | -0.006 (0.978) | 0.107 (0.612) |
| lh_parsopercularis | 0.102 (0.627) | -0.121 (0.566) | -0.115 (0.584) | 0.157 (0.454) | -0.020 (0.923) | 0.037 (0.859) |
| lh_parsorbitalis | -0.388 (0.055) | 0.325 (0.113) | -0.268 (0.195) | 0.072 (0.731) | -0.418 (0.038)***** | 0.233 (0.261) |
| lh_parstriangularis | -0.236 (0.255) | 0.211 (0.311) | 0.257 (0.214) | -0.053 (0.801) | 0.041 (0.847) | 0.084 (0.689) |
| lh_pericalcarine | 0.337 (0.099) | -0.243 (0.241) | 0.379 (0.062) | -0.389 (0.055) | 0.466 (0.019)***** | -0.401 (0.047)***** |
| lh_postcentral | -0.080 (0.704) | 0.189 (0.366) | 0.147 (0.483) | -0.165 (0.430) | 0.056 (0.790) | -0.004 (0.984) |
| lh_posteriorcingulate | -0.291 (0.159) | 0.279 (0.177) | 0.238 (0.253) | -0.286 (0.166) | -0.005 (0.981) | -0.035 (0.870) |
| lh_precentral | -0.329 (0.108) | 0.299 (0.147) | -0.096 (0.648) | 0.019 (0.930) | -0.262 (0.205) | 0.182 (0.384) |
| lh_precuneus | 0.221 (0.288) | -0.175 (0.403) | 0.257 (0.216) | -0.302 (0.142) | 0.311 (0.130) | -0.303 (0.140) |
| lh_rostralanteriorcingulate | -0.457 (0.022)***** | 0.450 (0.024)***** | -0.019 (0.927) | -0.063 (0.766) | -0.284 (0.168) | 0.213 (0.307) |
| lh_rostralmiddlefrontal | -0.222 (0.287) | 0.292 (0.156) | 0.467 (0.019)***** | -0.477 (0.016)***** | 0.197 (0.346) | -0.156 (0.457) |
| lh_superiorfrontal | -0.059 (0.780) | 0.081 (0.700) | -0.339 (0.097) | 0.336 (0.101) | -0.273 (0.186) | 0.273 (0.187) |
| lh_superiorparietal | 0.053 (0.800) | -0.077 (0.714) | 0.076 (0.717) | -0.157 (0.455) | 0.085 (0.686) | -0.149 (0.476) |
| lh_superiortemporal | -0.542 (0.005)****** | 0.312 (0.129) | -0.207 (0.321) | 0.206 (0.323) | -0.466 (0.019)* | 0.316 (0.124) |
| lh_supramarginal | -0.326 (0.112) | 0.095 (0.652) | 0.213 (0.307) | -0.256 (0.216) | -0.043 (0.838) | -0.119 (0.571) |
| lh_frontalpole | -0.231 (0.268) | 0.233 (0.263) | 0.334 (0.103) | -0.309 (0.133) | 0.098 (0.64) | -0.077 (0.716) |
| lh_temporalpole | -0.148 (0.480) | -0.104 (0.622) | 0.156 (0.457) | -0.360 (0.077) | 0.022 (0.917) | -0.302 (0.142) |
| lh_transversetemporal | -0.18 (0.390) | 0.121 (0.566) | -0.141 (0.501) | 0.131 (0.531) | -0.206 (0.324) | 0.157 (0.453) |
| lh_insula | -0.231 (0.266) | 0.105 (0.617) | 0.116 (0.581) | -0.139 (0.508) | -0.055 (0.792) | -0.034 (0.872) |

The number represents the corresponding test (fe-fe1 is the result from Test 1 and fear2 represent the results from Test 2 and etc.); fear_mean and fe-su_mean stands for the average of the results. * indicates p < 0.05, and ** indicates p < 0.01.

**Table S5.** The correlation coefficients for the relationship between the volume of the right hemispheric regions and fear-fear (fear-surprise) choice in Test 1, Test 2 and the average of two tests.

| Right Hemisphere | fe-fe1 | fe-su1 | fe-fe2 | fe-su2 | fear_mean | fe-su_mean |
| --- | --- | --- | --- | --- | --- | --- |
| rh_bankssts | -0.144 (0.492) | 0.151 (0.471) | 0.089 (0.673) | -0.036 (0.864) | -0.023 (0.914) | 0.061 (0.771) |
| rh_caudalanteriorcingulate | -0.080 (0.705) | 0.372 (0.067) | -0.160 (0.445) | 0.206 (0.322) | -0.160 (0.446) | 0.350 (0.086) |
| rh_caudalmiddlefrontal | -0.528 (0.007)** | 0.268 (0.195) | 0.123 (0.558) | -0.144 (0.492) | -0.227 (0.276) | 0.055 (0.794) |
| rh_cuneus | -0.175 (0.403) | 0.316 (0.124) | 0.226 (0.278) | -0.221 (0.288) | 0.055 (0.794) | 0.030 (0.886) |
| rh_entorhinal | -0.155 (0.460) | 0.038 (0.856) | -0.156 (0.456) | 0.039 (0.854) | -0.202 (0.334) | 0.048 (0.820) |
| rh_fusiform | -0.126 (0.549) | 0.033 (0.874) | -0.011 (0.958) | 0.026 (0.902) | -0.082 (0.695) | 0.036 (0.863) |
| rh_inferiorparietal | -0.444 (0.026)* | 0.372 (0.067) | 0.296 (0.151) | -0.28 (0.175) | -0.055 (0.792) | 0.022 (0.916) |
| rh_inferiortemporal | -0.446 (0.025)* | 0.257 (0.215) | 0.032 (0.880) | -0.126 (0.549) | -0.242 (0.244) | 0.061 (0.773) |
| rh_isthmuscingulate | -0.241 (0.245) | 0.216 (0.300) | 0.329 (0.108) | -0.281 (0.174) | 0.089 (0.674) | -0.067 (0.750) |
| rh_lateraloccipital | -0.081 (0.701) | -0.028 (0.893) | 0.179 (0.391) | -0.263 (0.203) | 0.078 (0.711) | -0.194 (0.353) |
| rh_lateralorbitofrontal | -0.150 (0.474) | 0.083 (0.693) | -0.101 (0.631) | -0.045 (0.830) | -0.160 (0.445) | 0.017 (0.937) |
| rh_lingual | -0.084 (0.690) | 0.209 (0.315) | 0.229 (0.271) | -0.154 (0.463) | 0.111 (0.597) | 0.015 (0.943) |
| rh_medialorbitofrontal | -0.249 (0.230) | 0.335 (0.102) | -0.034 (0.872) | 0.102 (0.626) | -0.171 (0.412) | 0.259 (0.211) |
| rh_middletemporal | -0.384 (0.058) | 0.257 (0.215) | 0.116 (0.580) | -0.128 (0.542) | -0.146 (0.487) | 0.060 (0.777) |
| rh_parahippocampal | -0.518 (0.008)** | 0.400 (0.048)***** | -0.048 (0.820) | 0.018 (0.932) | -0.34 (0.096) | 0.239 (0.249) |
| rh_paracentral | 0.029 (0.890) | 0.136 (0.516) | -0.022 (0.915) | 0.024 (0.908) | 0.001 (0.994) | 0.094 (0.656) |
| rh_parsopercularis | -0.094 (0.657) | -0.150 (0.474) | 0.331 (0.106) | -0.376 (0.064) | 0.177 (0.397) | -0.339 (0.097) |
| rh_parsorbitalis | -0.275 (0.184) | 0.283 (0.170) | 0.046 (0.828) | -0.096 (0.647) | -0.130 (0.534) | 0.096 (0.649) |
| rh_parstriangularis | -0.335 (0.102) | 0.250 (0.228) | 0.191 (0.360) | -0.233 (0.263) | -0.064 (0.762) | -0.015 (0.943) |
| rh_pericalcarine | 0.117 (0.577) | -0.102 (0.627) | 0.340 (0.096) | -0.394 (0.051) | 0.309 (0.133) | -0.324 (0.114) |
| rh_postcentral | -0.238 (0.253) | 0.208 (0.319) | 0.078 (0.712) | -0.149 (0.476) | -0.086 (0.682) | 0.017 (0.936) |
| rh_posteriorcingulate | -0.027 (0.898) | 0.134 (0.523) | 0.063 (0.763) | -0.212 (0.310) | 0.029 (0.892) | -0.067 (0.751) |
| rh_precentral | -0.084 (0.691) | 0.003 (0.989) | -0.035 (0.868) | -0.014 (0.947) | -0.074 (0.725) | -0.008 (0.970) |
| rh_precuneus | 0.162 (0.438) | -0.211 (0.311) | 0.189 (0.367) | -0.256 (0.216) | 0.229 (0.272) | -0.293 (0.155) |
| rh_rostralanteriorcingulate | -0.141 (0.501) | 0.316 (0.123) | -0.008 (0.968) | 0.021 (0.920) | -0.090 (0.670) | 0.194 (0.353) |
| rh_rostralmiddlefrontal | -0.311 (0.130) | 0.346 (0.090) | 0.113 (0.591) | -0.135 (0.52) | -0.105 (0.618) | 0.105 (0.616) |
| rh_superiorfrontal | -0.012 (0.954) | -0.030 (0.887) | 0.039 (0.853) | -0.140 (0.503) | 0.020 (0.923) | -0.112 (0.595) |
| rh_superiorparietal | -0.055 (0.794) | -0.047 (0.822) | 0.180 (0.389) | -0.251 (0.226) | 0.094 (0.655) | -0.196 (0.347) |
| rh_superiortemporal | -0.353 (0.084) | 0.253 (0.222) | -0.110 (0.599) | 0.145 (0.488) | -0.286 (0.165) | 0.242 (0.244) |
| rh_supramarginal | -0.174 (0.405) | 0.172 (0.411) | -0.175 (0.402) | 0.119 (0.570) | -0.226 (0.277) | 0.178 (0.394) |
| rh_frontalpole | -0.410 (0.042)* | 0.483 (0.014)***** | -0.052 (0.805) | 0.128 (0.543) | -0.279 (0.176) | 0.360 (0.077) |
| rh_temporalpole | 0.141 (0.501) | -0.167 (0.426) | 0.267 (0.196) | -0.256 (0.217) | 0.271 (0.189) | -0.267 (0.197) |
| rh_transversetemporal | -0.157 (0.452) | 0.209 (0.317) | 0.106 (0.613) | -0.016 (0.941) | -0.019 (0.930) | 0.108 (0.608) |
| rh_insula | -0.110 (0.602) | 0.058 (0.782) | 0.016 (0.938) | -0.128 (0.542) | -0.053 (0.800) | -0.053 (0.800) |

**Table S6.** The correlation coefficients for the relationship between the volume of the subcortical regions and fear-fear (fear-surprise) choice in Test 1, Test 2 and the average of two tests.

| Subcortical | fe-fe1 | fe-su1 | fe-fe2 | fe-su2 | fear_mean | fe-su_mean |
| --- | --- | --- | --- | --- | --- | --- |
| Left_cerebellum | -0.266 (0.199) | 0.203 (0.331) | -0.013 (0.951) | -0.041 (0.847) | -0.167 (0.426) | 0.088 (0.677) |
| Left_thalamusproper | -0.393 (0.052) | 0.375 (0.064) | -0.037 (0.859) | 0.022 (0.917) | -0.259 (0.211) | 0.228 (0.273) |
| Left_caudate | -0.011 (0.959) | 0.051 (0.808) | 0.141 (0.502) | -0.053 (0.801) | 0.093 (0.660) | -0.007 (0.974) |
| Left_putamen | 0.021 (0.922) | -0.158 (0.450) | -0.340 (0.096) | 0.182 (0.384) | -0.227 (0.275) | 0.033 (0.876) |
| Left_pallidum | 0.293 (0.155) | -0.458 (0.021)* | -0.028 (0.894) | -0.163 (0.437) | 0.154 (0.463) | -0.370 (0.069) |
| Left_hippocampus | -0.360 (0.077) | 0.299 (0.147) | -0.194 (0.352) | 0.217 (0.297) | -0.350 (0.086) | 0.316 (0.124) |
| Left_amygdala | -0.606 (0.001)****** | 0.571 (0.003)****** | -0.432 (0.031)***** | 0.445 (0.026)***** | -0.663 (0.000)****** | 0.625 (0.001)****** |
| Left_accumbens | -0.107 (0.610) | -0.070 (0.740) | -0.115 (0.586) | 0.034 (0.871) | -0.144 (0.492) | -0.017 (0.937) |
| Right_cerebellum- | -0.223 (0.283) | 0.086 (0.682) | -0.055 (0.795) | -0.079 (0.708) | -0.171 (0.414) | -0.004 (0.984) |
| Right_thalamusproper | -0.124 (0.554) | 0.044 (0.836) | -0.125 (0.553) | 0.038 (0.856) | -0.161 (0.441) | 0.051 (0.810) |
| Right_caudate | 0.030 (0.888) | 0.007 (0.975) | 0.154 (0.462) | -0.050 (0.811) | 0.126 (0.549) | -0.030 (0.886) |
| Right_putamen | -0.010 (0.961) | -0.180 (0.390) | -0.168 (0.422) | 0.026 (0.900) | -0.124 (0.554) | -0.084 (0.689) |
| Right_pallidum | 0.018 (0.933) | -0.150 (0.476) | 0.110 (0.600) | -0.193 (0.355) | 0.088 (0.676) | -0.215 (0.301) |
| Right_hippocampus | -0.278 (0.178) | 0.231 (0.267) | -0.116 (0.581) | 0.079 (0.709) | -0.246 (0.235) | 0.184 (0.378) |
| Right_amygdala | -0.270 (0.192) | 0.051 (0.808) | -0.302 (0.142) | 0.271 (0.191) | -0.372 (0.067) | 0.212 (0.310) |
| Right_accumbens | -0.107 (0.610) | 0.033 (0.875) | 0.090 (0.670) | -0.110 (0.599) | 0.000 (0.998) | -0.056 (0.791) |

**Table S7.** The subcortical volume (mm^3^) of the thirty participants in Scan 1 (left hemisphere).

| Subject | Left-Cerebellum-Cortex | Left-Thalamus-Proper | Left-Caudate | Left-Putamen | Left-Pallidum | Left-Hippocampus | Left-Amygdala |
| --- | --- | --- | --- | --- | --- | --- | --- |
| 1 | 45335 | 6473 | 3522 | 5785 | 1521 | 4757 | 1840 |
| 2 | 52016 | 6879 | 3733 | 7199 | 2023 | 4316 | 1761 |
| 3 | 49028 | 6580 | 3342 | 6319 | 1688 | 3942 | 1393 |
| 4 | 48967 | 7309 | 3676 | 6770 | 1497 | 4046 | 1947 |
| 5 | 43467 | 5633 | 3083 | 5503 | 1620 | 3611 | 1459 |
| 6 | 54939 | 7724 | 4293 | 8266 | 2470 | 4911 | 2076 |
| 7 | 57903 | 7809 | 3558 | 7204 | 2041 | 5044 | 1706 |
| 8 | 43852 | 6266 | 3727 | 6987 | 2025 | 3607 | 1410 |
| 9 | 47873 | 6672 | 3978 | 6918 | 1721 | 4331 | 1895 |
| 10 | 51766 | 7089 | 3155 | 6014 | 1778 | 4030 | 1694 |
| 11 | 49269 | 6723 | 4768 | 7683 | 1941 | 4841 | 1542 |
| 12 | 42944 | 6437 | 2969 | 7134 | 1777 | 3773 | 1565 |
| 13 | 42033 | 6446 | 4000 | 6907 | 1960 | 3717 | 1420 |
| 14 | 57259 | 7488 | 4380 | 6624 | 1997 | 4211 | 2157 |
| 15 | 50391 | 6761 | 3757 | 6254 | 1733 | 3786 | 1629 |
| 16 | 42498 | 6268 | 3966 | 6763 | 1921 | 3757 | 1650 |
| 17 | 55472 | 8675 | 4019 | 7233 | 2111 | 4353 | 1772 |
| 18 | 47667 | 6717 | 2936 | 6630 | 1717 | 3927 | 1443 |
| 19 | 48960 | 5987 | 3473 | 6753 | 1811 | 3786 | 1614 |
| 20 | 52428 | 6860 | 3546 | 6080 | 1920 | 4476 | 1821 |
| 21 | 48524 | 6535 | 3205 | 6172 | 1885 | 4347 | 1507 |
| 22 | 55047 | 7975 | 4151 | 8025 | 1973 | 4693 | 2353 |
| 23 | 44364 | 6543 | 3463 | 6261 | 1724 | 3988 | 1491 |
| 24 | 45785 | 7185 | 4275 | 7622 | 1961 | 4358 | 2004 |
| 25 | 55889 | 7442 | 3744 | 6515 | 1866 | 4266 | 1654 |
| 26 | 48536 | 7420 | 4342 | 7960 | 2173 | 4176 | 1594 |
| 27 | 56226 | 6625 | 3595 | 5944 | 1784 | 3952 | 1598 |
| 28 | 53237 | 7071 | 3392 | 7666 | 2194 | 4639 | 1662 |
| 29 | 58822 | 7601 | 4744 | 7008 | 2070 | 4254 | 2047 |
| 30 | 46994 | 6126 | 3613 | 6529 | 1822 | 3854 | 1778 |

**Table S8.** The subcortical volume (mm^3^) of the thirty participants in Scan 1 (right hemisphere).

| Subject | Right-Thalamus-Proper | Right-Caudate | Right-Putamen | Right-Pallidum | Right-Hippocampus | Right-Amygdala | Right-Accumbens-area |
| --- | --- | --- | --- | --- | --- | --- | --- |
| 1 | 6351 | 3623 | 5546 | 1626 | 4602 | 1761 | 666 |
| 2 | 7979 | 3793 | 7209 | 1816 | 4740 | 1934 | 728 |
| 3 | 6040 | 3459 | 6526 | 1596 | 4213 | 1430 | 617 |
| 4 | 6524 | 3770 | 6462 | 1560 | 4368 | 2277 | 777 |
| 5 | 6033 | 3027 | 5323 | 1435 | 3798 | 1531 | 627 |
| 6 | 8163 | 3967 | 7934 | 1945 | 5191 | 2213 | 791 |
| 7 | 7597 | 3666 | 7075 | 1796 | 4965 | 1743 | 771 |
| 8 | 6162 | 3915 | 6601 | 1804 | 3720 | 1675 | 843 |
| 9 | 6664 | 4262 | 6302 | 1694 | 4300 | 1887 | 717 |
| 10 | 7630 | 3352 | 5991 | 1660 | 4249 | 1670 | 634 |
| 11 | 7314 | 5027 | 7423 | 1726 | 4862 | 1955 | 900 |
| 12 | 6327 | 3735 | 6837 | 1614 | 3834 | 1710 | 725 |
| 13 | 6568 | 4250 | 6280 | 1606 | 3813 | 1523 | 667 |
| 14 | 7552 | 4332 | 6535 | 1892 | 4568 | 2052 | 906 |
| 15 | 6989 | 3400 | 5777 | 1553 | 3721 | 1664 | 695 |
| 16 | 6715 | 4095 | 6120 | 1647 | 4006 | 1794 | 630 |
| 17 | 8557 | 4271 | 6556 | 1869 | 4347 | 1823 | 751 |
| 18 | 6910 | 3225 | 6401 | 1688 | 4010 | 1538 | 619 |
| 19 | 6003 | 3584 | 6256 | 1427 | 3857 | 1688 | 683 |
| 20 | 8077 | 3534 | 6143 | 1609 | 4613 | 1915 | 642 |
| 21 | 6791 | 3072 | 6072 | 1845 | 4132 | 1636 | 670 |
| 22 | 7875 | 4225 | 7753 | 1820 | 4810 | 2467 | 967 |
| 23 | 6692 | 3458 | 5963 | 1583 | 4129 | 1633 | 662 |
| 24 | 7222 | 4531 | 6967 | 1706 | 4498 | 2317 | 719 |
| 25 | 7457 | 3973 | 6241 | 1683 | 4441 | 1733 | 712 |
| 26 | 7473 | 4375 | 7584 | 1850 | 4177 | 1796 | 961 |
| 27 | 6887 | 3595 | 5820 | 1477 | 4082 | 1526 | 571 |
| 28 | 7756 | 3439 | 6684 | 1673 | 4505 | 1876 | 710 |
| 29 | 7923 | 4734 | 6588 | 1958 | 4372 | 2260 | 838 |
| 30 | 6314 | 3921 | 6475 | 1586 | 3750 | 1530 | 801 |

**Table S9.** The subcortical volume (mm^3^) of the thirty participants in Scan 2 (left hemisphere).

| Subject | Left-Cerebellum-Cortex | Left-Thalamus-Proper | Left-Caudate | Left-Putamen | Left-Pallidum | Left-Hippocampus | Left-Amygdala |
| --- | --- | --- | --- | --- | --- | --- | --- |
| 1 | 45673 | 6266 | 3535 | 5719 | 1679 | 4859 | 1967 |
| 2 | 52698 | 8083 | 3633 | 7098 | 1983 | 4488 | 1719 |
| 3 | 48830 | 7649 | 3299 | 6184 | 1036 | 3781 | 1390 |
| 4 | 48944 | 6811 | 3673 | 6618 | 1622 | 4133 | 2129 |
| 5 | 45706 | 5826 | 2760 | 5365 | 1610 | 3867 | 1479 |
| 6 | 56557 | 8847 | 4027 | 8053 | 2116 | 5153 | 2009 |
| 7 | 56562 | 7362 | 3852 | 6991 | 1968 | 4449 | 1836 |
| 8 | 45208 | 6681 | 3752 | 6916 | 1956 | 3714 | 1559 |
| 9 | 47740 | 6213 | 4275 | 6676 | 1960 | 4121 | 1805 |
| 10 | 52030 | 7377 | 2856 | 5858 | 1792 | 4008 | 1599 |
| 11 | 49806 | 7060 | 4493 | 7257 | 1940 | 4948 | 1533 |
| 12 | 44218 | 6726 | 3008 | 7037 | 1758 | 4054 | 1509 |
| 13 | 42914 | 7033 | 4055 | 7039 | 1789 | 3910 | 1436 |
| 14 | 58579 | 8377 | 4370 | 6593 | 1826 | 4343 | 2017 |
| 15 | 52104 | 6725 | 3503 | 6384 | 1623 | 4188 | 1648 |
| 16 | 39787 | 6578 | 3919 | 6661 | 1683 | 3833 | 1865 |
| 17 | 56993 | 8544 | 3953 | 7067 | 2016 | 4554 | 1627 |
| 18 | 48020 | 6626 | 3068 | 6656 | 1838 | 3836 | 1552 |
| 19 | 47712 | 6029 | 3109 | 6294 | 1732 | 3546 | 1621 |
| 20 | 52487 | 7217 | 3504 | 6077 | 1733 | 4562 | 1791 |
| 21 | 48122 | 6986 | 3329 | 6106 | 1881 | 4496 | 1640 |
| 22 | 55016 | 8439 | 3974 | 7923 | 1783 | 4763 | 2149 |
| 23 | 44928 | 6940 | 3432 | 6350 | 1750 | 3970 | 1454 |
| 24 | 45648 | 6621 | 4441 | 7375 | 1901 | 4537 | 1919 |
| 25 | 57296 | 7829 | 3808 | 6528 | 1878 | 4010 | 1497 |
| 26 | 50792 | 7896 | 4239 | 7863 | 2109 | 4350 | 1851 |
| 27 | 55194 | 6214 | 3556 | 6148 | 1765 | 4122 | 1795 |
| 28 | 53694 | 6469 | 3375 | 7055 | 2120 | 4276 | 1754 |
| 29 | 57325 | 7709 | 4764 | 6859 | 2048 | 4203 | 2031 |
| 30 | 45702 | 5722 | 3667 | 6653 | 1871 | 3765 | 1604 |

**Table S10.** The The subcortical volume (mm^3^) of the thirty participants in Scan 2 (right hemisphere).

| Subject | Right-Thalamus-Proper | Right-Caudate | Right-Putamen | Right-Pallidum | Right-Hippocampus | Right-Amygdala | Right-Accumbens-area |
| --- | --- | --- | --- | --- | --- | --- | --- |
| 1 | 6528 | 3551 | 5750 | 1494 | 4805 | 1706 | 681 |
| 2 | 7888 | 3708 | 6816 | 1814 | 4660 | 1744 | 651 |
| 3 | 6231 | 3335 | 6098 | 1568 | 3920 | 1444 | 616 |
| 4 | 6197 | 3785 | 6193 | 1704 | 4102 | 1729 | 842 |
| 5 | 5827 | 2982 | 4967 | 1459 | 4010 | 1559 | 631 |
| 6 | 8376 | 3654 | 7845 | 2118 | 5035 | 2034 | 629 |
| 7 | 7168 | 3583 | 7026 | 1776 | 4638 | 1832 | 775 |
| 8 | 6423 | 4062 | 6934 | 1740 | 3663 | 1849 | 820 |
| 9 | 6462 | 4503 | 6284 | 1604 | 4253 | 2060 | 776 |
| 10 | 7427 | 3092 | 5965 | 1692 | 4322 | 1705 | 613 |
| 11 | 6933 | 4993 | 6935 | 1747 | 4745 | 1591 | 876 |
| 12 | 6283 | 3702 | 6669 | 1522 | 4079 | 1608 | 660 |
| 13 | 6893 | 4364 | 6783 | 1790 | 4133 | 1519 | 696 |
| 14 | 7842 | 4390 | 6435 | 1823 | 4678 | 1951 | 912 |
| 15 | 6881 | 3176 | 6055 | 1483 | 3832 | 1681 | 710 |
| 16 | 6494 | 4112 | 6314 | 1637 | 3996 | 2103 | 681 |
| 17 | 8371 | 4026 | 6583 | 1874 | 4438 | 1787 | 738 |
| 18 | 6855 | 3264 | 6462 | 1573 | 4051 | 1610 | 673 |
| 19 | 6001 | 3145 | 6100 | 1457 | 3790 | 1628 | 626 |
| 20 | 7716 | 3560 | 6078 | 1685 | 4690 | 1793 | 611 |
| 21 | 6784 | 3530 | 6168 | 1871 | 4125 | 1689 | 711 |
| 22 | 7544 | 4206 | 7994 | 1870 | 4861 | 2341 | 1048 |
| 23 | 7241 | 3118 | 5932 | 1436 | 4021 | 1564 | 610 |
| 24 | 7110 | 4442 | 7108 | 1708 | 4823 | 1781 | 743 |
| 25 | 7257 | 4029 | 6415 | 1563 | 4344 | 1509 | 711 |
| 26 | 7301 | 4233 | 7284 | 1848 | 4256 | 1840 | 882 |
| 27 | 6732 | 3509 | 5925 | 1336 | 4335 | 1863 | 586 |
| 28 | 7314 | 3433 | 6675 | 1713 | 4418 | 1515 | 756 |
| 29 | 7935 | 4708 | 6466 | 1896 | 4480 | 1994 | 751 |
| 30 | 6427 | 3687 | 6764 | 1595 | 3714 | 1708 | 788 |
